# Antimicrobial and Antiviral Properties of Triclosan-Containing Polymer Composite: Aging Effects of pH, UV, and Sunlight Exposure

**DOI:** 10.3390/polym15051236

**Published:** 2023-02-28

**Authors:** Zhandos Tauanov, Olzhas Zakiruly, Zhuldyz Baimenova, Alzhan Baimenov, Nuraly S. Akimbekov, Dmitriy Berillo

**Affiliations:** 1 Faculty of Chemistry and Chemical Technology, Al-Farabi Kazakh National University, Almaty 050040, Kazakhstan; 2 Department of Research and Development, LLP “Marmar Kazakhstan”, Taldykorgan 040008, Kazakhstan; 3 Laboratory of Green Energy and Environment, National Laboratory Astana, Nazarbayev University, Astana 010000, Kazakhstan; 4 Department of Biotechnology, Al-Farabi Kazakh National University, Almaty 050040, Kazakhstan; 5 Department of Chemistry and Biochemical Engineering, Institute of Chemical and Biological Technologies, Satbayev University, Almaty 050013, Kazakhstan

**Keywords:** polymer, composite, triclosan, antimicrobial, antiviral, coronavirus

## Abstract

The present study deals with the synthesis and characterization of a polymer composite based on an unsaturated ester loaded with 5 wt.% triclosan, produced by co-mixing on an automated hardware system. The polymer composite’s non-porous structure and chemical composition make it an ideal material for surface disinfection and antimicrobial protection. According to the findings, the polymer composite effectively inhibited (100%) the growth of *Staphylococcus aureus* 6538-P under exposure to physicochemical factors, including pH, UV, and sunlight, over a 2-month period. In addition, the polymer composite demonstrated potent antiviral activity against human influenza virus strain A and the avian coronavirus infectious bronchitis virus (IBV), with infectious activities of 99.99% and 90%, respectively. Thus, the resulting triclosan-loaded polymer composite is revealed to have a high potential as a surface-coating non-porous material with antimicrobial properties.

## 1. Introduction

Infectious diseases result in 10 million deaths every year, accounting for almost 20% of all deaths worldwide, and data indicate that approximately 80% of human infections arise from contaminated surfaces [1,2]. World Health Organization (WHO) data show that 550 million people get sick each year, and 230,000 of them die because of diarrheal diseases caused by foodborne pathogens. Contamination of the public water supply, poor sanitation, and unhygienic practices cause additional 842,000 deaths from diarrhea in low-income and average-income countries [3]. Moreover, the new, highly contagious SARS-CoV-2 virus, disseminated on airborne droplets and deposited on various surfaces, has led to more than 643 million cases and 6.6 million deaths, according to the WHO (9 December 2022) [4]. Riddell et al. demonstrated that under controlled conditions (20 °C and 50% relative humidity), infectious SARS-CoV-2 remains viable for at least 28 days on non-porous surfaces (stainless steel, glass, vinyl, polymer, and paper) [5]. The survival of SARS-CoV-2 on hospital protective clothing was observed up to 21 days after its inoculation on plastic materials and N95 masks [6]. Long-life microbial contamination makes the development of antimicrobial surfaces vital.

Therefore, extensive research has been conducted to improve antimicrobial surfaces, which can prevent pathogen attachment and subsequent contamination [7,8,9]. Solid surfaces can serve as a matrix for immobilizing active biocidal agents using various methods, such as chemical grafting [10], layer-by-layer self-assembly, surface impregnation [11], and physical entrapment [12]. Different inorganic substances (heavy metals and titanium oxide [13]) and organic compounds (various antibiotics [12], quaternary ammonium disinfectants [14,15], and phenol derivatives [16]) are commonly used for immobilization to obtain effective antibacterial surfaces. Depending on the mechanism of antibacterial action, such surface-modified composites can be divided into three classes: anti-adhesive surfaces, bactericidal surfaces, and the surface release of biocide agents [17].

The direct doping of antimicrobial agents into bulk materials is one of the most effective methods since these materials can work through contact destruction and the long-term release of antimicrobial agents. In contrast, surface-modified coatings are not always beneficial for long-term stability due to the degradation and peeling of coatings. Ideal antibacterial materials should offer clear advantages, such as broad-spectrum bactericidal efficacy, biocompatibility, low toxicity, and reasonable cost [18]. Based on these parameters, polymeric antimicrobial materials and their composites are generally preferable to bare metal nanoparticles [19,20]. Polymeric antibacterial materials of natural origin (chitosan and starch) and synthetic polymers (polybiguanide, quaternary ammonium salts, triclosan, and N-halamine) can have antibacterial properties on their own or can be modified with inorganic metal nanoparticles, including TiO_2_, ZnO, CuO, and silver [21]. Triclosan represents halogenated phenoxyphenols and has an aromatic compound with ethers, phenols, and chlorine in its structure [22]. Even at low concentrations, triclosan exhibits a broad spectrum of antibacterial and antifungal activities due to its destructive effect on bacterial enzymes responsible for cell wall synthesis. In contrast, at high concentrations, triclosan disrupts the bacterial membrane integrity, causing their death [23]. Iyigundogdu and colleagues modified cotton fabrics with 7% glucapon, 3% sodium pentaborate pentahydrate, and 0.03% triclosan. They reported that after 2 h of contact with feline coronavirus, the virus concentration was reduced by 94% [24]. In another research study, Orhan tested poly(ethylene terephthalate) (PET) and cotton surfaces modified with triclosan for antibacterial properties against *S. aureus* and *Escherichia coli* [25]. They reported that after 3 h of contact, PET/triclosan reduced *S. aureus* growth by 99.94% and *E. coli* growth by 99.45%. In addition, the bactericidal efficiency after 10 and 20 cycles of washing decreased to 91.6% and 70.99 for *S. aureus* and 87.91 and 69.23% for *E. coli*, respectively. In this regard, polymer scaffolds with greater drug retention can reduce the leaching of triclosan and maintain its chemical structure so it is accessible for interactions with bacteria over time. Table 1 presents the results of several studies related to the application of antimicrobial agents to modify the polymeric matrix.

This study continues our previous study on the production of a polymeric composite with a non-porous structure from an unsaturated ester of orthophthalic acid loaded with the antimicrobial agent triclosan. The obtained reference and triclosan-loaded polymeric composites were characterized using XRF, BET, TGA, and SEM-EDS to retrieve data on the elemental composition, morphology, and thermal properties of the materials. Furthermore, to study the aging effects of the antimicrobial polymer composite, it was exposed to UV, pH, and sunlight and then tested using *S. aureus* ATCC 6538-P, broadly conserved among Gram-positive bacteria. In addition, antiviral activity tests were performed using the human influenza virus, strain A/WKO/46/19, and chicken infectious bronchitis virus (IBV) to demonstrate the antimicrobial properties of the polymer composite against viruses commonly dispersed on surfaces.

## 2. Materials and Methods

### 2.1. Materials

The following chemicals were purchased from AppliChem, Darmstadt, Germany, to produce polymer composites with and without loading the antimicrobial agent: methyl ethyl ketone peroxide (99%), unsaturated polyester of orthophthalic acid (99%), triclosan (5-chloro-2-(2,4-dichloro-phenoxy)phenol) (99.5 wt.%), and CaCO_3_ (95 wt.%). The reference (without triclosan) and experimental (with 5 wt.% triclosan) samples of the polymeric composites were analyzed in triplicate, and the average characteristics of the synthesis are presented. The culture media (nutrient agar, nutrient broth, and tryptic soy broth) used for the antimicrobial experiments were purchased from Himedia, India. Lecithin (phosphatidylcholine min. 60%; *E. coli:* negative; iodine number: 60–70; peroxide number: max. 3.0; lysophosphatidylcholine: max. 3%; non-polar lipids: max. 20%; distilled water (GOST R 58144-2018): max. 2%) and tween-80 (MW 1310 g/mol) were purchased from AppliChem, Germany. Ethanol (96%) was purchased from Talgar Spirt (GOST 5962-2013), Almaty, Kazakhstan. Experiments on the effects of pH, UV, and sunlight exposure were performed using *S. aureus* ATCC 6538-P, a test strain obtained from the Republican Collection of Microorganisms, Astana, Kazakhstan. Two model strains of viruses were used to study the antiviral activity: orthomyxovirus, human influenza virus, strain A/WKO/46/19 (pandemic version from the Republic of Kazakhstan in 2019), initial concentration 10^7^ EID/1 mL), and avian coronavirus infectious bronchitis virus (IBV) (strain H-120, Massachusetts serotype), vaccine strain, initial concentration 10^4^ EID/1 mL. The reagents used for the antiviral test activity of the polymer composite with 5 wt.% triclosan include tablets for the preparation of pH 7.3–7.5 phosphate-buffered saline (Amresco, Boise, ID, USA), Pure Link Viral DNA/RNA kit, M-MLV reverse transcriptase (Invitrogen, Waltham, MA, USA) and reverse transcriptase buffer (Invitrogen, Waltham, MA, USA), and Maxima SYBR Green qPCR Master Mix, (ThermoFisher Scientific, Waltham, MA, USA). All chemicals used were of analytical grade and were used as supplied.

### 2.2. Preparation of Polymer Composites with Triclosan

**Figure 1:**
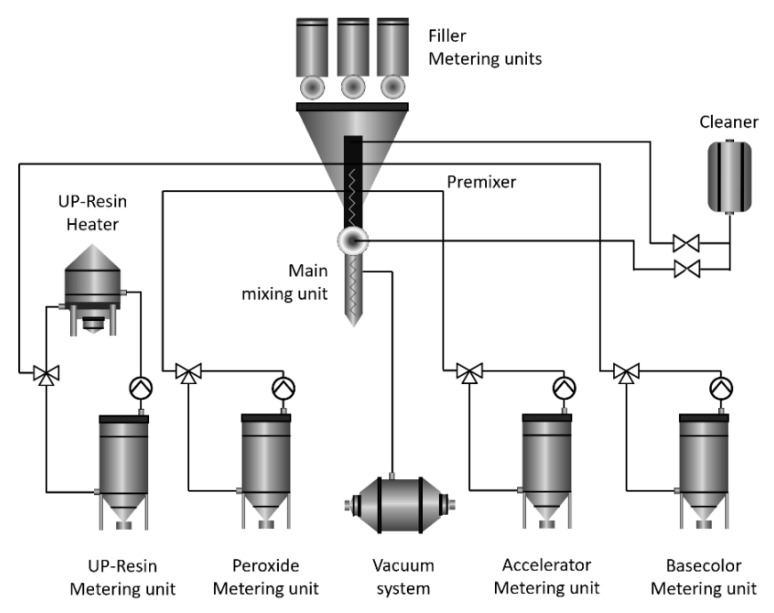
Manufacturing process flow of the polymer composite.

An advanced automated technological complex (RESPECTA, Wulfrath, Germany) was applied to manufacture the polymer composites used in this study, as shown in Figure 1. The main units of the complex that allow the precise and safe production of polymeric composites are filler metering units, a cleaner, a resin heater, a metering unit, mixing and premixing units, a metering unit, and a vacuum system, as well as peroxide, accelerator, and base color metering units.

A control polymer composite without the addition of triclosan was manufactured using an unsaturated polyester of orthophthalic acid as a binder, calcium carbonate (CaCO_3_), and silicon dioxide (SiO_2_) fillers. The hardener (catalyst) was methyl ethyl ketone peroxide (MEKP), according to a previously published procedure [35]. The sample of the polymer composite with 5 wt.% triclosan was manufactured following the same procedure but with a reduced amount of silicon dioxide, as described in our previous publication [36]. The chemical compositions of the reference and polymer composites used for preparation are shown in Table 2.

### 2.3. Characterization of Polymer Composite

The polymer composites were characterized using advanced methods. First, the chemical compositions of the reference and triclosan-loaded polymeric composites were quantified by standardless X-ray Fluorescence (Malvern Panalytical, Cambridge, UK). The elemental analysis was performed in a vacuum at 40–50 kV by using anhydrous boric acid as a flux to prepare pellets of the reference and triclosan-loaded samples in a 1/3 ratio. The thermal properties (50 °C to 700 °C, 10 °C min heating rate) of the polymeric composites were studied on a Simultaneous Thermal Analyzer (STA6000, Perkin Elmer, Waltham, MA, USA) under a nitrogen atmosphere. The surface morphology and spot elemental analyses of the polymeric composites were performed using a Scanning Electron Microscope (JEOL 6380 LV, LV mode, at 20 KV) equipped with a backscattered electron detector (JEOL, Tokyo, Japan), and a Si(Li) Energy-Dispersive X-ray Spectrometer (INCA X-sight, Oxford Instruments, Abingdon, UK). The porous structure was determined through low-temperature nitrogen adsorption measured on an Autosorb-1 porosimeter (Quantachrome, Hook, UK). Finally, the specific surface area was calculated based on the BET model. The details of these and additional characterization methods applied for the polymeric composites are described elsewhere [36].

### 2.4. Antimicrobial Activity Tests of Polymer Composites

#### 2.4.1. Preparation of Test Samples

The objective was to evaluate the retention of the antimicrobial efficacy of a triclosan-containing composite sample after prolonged exposure to certain physicochemical factors. Accordingly, the samples were divided into 3 experimental groups. Preparation for the experiment was conducted as follows:

Group 1—the effect of the influence of sunlight: the sample was stored in natural light conditions under direct sunlight.

Group 2—the effect of UV irradiation: the sample was placed daily in a laminar box and exposed to UV irradiation (wavelength 254 nm); the exposure time was 2 h.

Group 3—the effect of the medium pH index: the samples were treated daily with buffer solutions with pH 2.0 (acidic medium) and 8.0 (basic medium) for 5 min, after which they were washed once with sterile distilled water.

Control and experimental composite samples were placed in individual sterile Petri dishes prior to the experiment. In parallel, 40 × 40 (±2.0) mm polyethylene cover films were prepared. All samples and cover films were sterilized with a 70% ethanol solution.

#### 2.4.2. Preparation of Test Culture Suspension

In this study, 18–24 h test cultures were used. An aliquot of the test strain was added to a test tube containing 5–6 mL 1/50 nutrient broth and homogenized. According to the McFarland standard, the optical density was 1.2 units, corresponding to a cell concentration of 6 × 10^8^ CFU/mL for bacterial suspensions. The estimated concentration of bacterial cells was prepared by performing 10-fold serial dilutions until a final inoculum of 6 × 10^5^ CFU/mL was obtained.

#### 2.4.3. Inoculation

**Figure 2:**
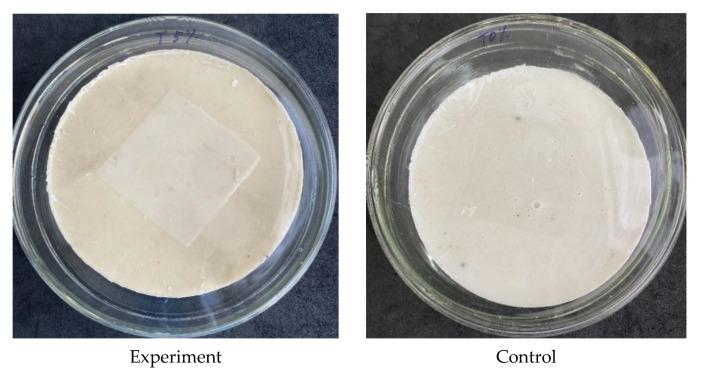
Inoculated experimental (with 5 wt.% triclosan) and control (without triclosan) composite samples covered with the release liner.

First, 0.4 mL of the culture suspension was added to the surfaces of the experimental and control samples and then immediately covered with a release liner (Figure 2), after which they were incubated for the appropriate exposure time. The details of the incubation parameters are presented in Table 3.

#### 2.4.4. Washing Samples

After incubation, the control and experimental samples were washed with tryptone-soy broth as a neutralizer with the addition of lecithin (1 g/L) and tween-80 (7 g/L). Washing was conducted by adding 10 mL of the neutralizer to the sample and removing the culture from the sample surface. The application of the neutralizing agent is vital in order to prevent false-positive results due to residual amounts of the active substance in the samples.

#### 2.4.5. Inoculation and Enumeration of Bacteria

In order to count colony-forming units (CFU), 10-fold serial dilutions (1/10, 1/100) were prepared from each sample, after which deep inoculation was performed. Plates containing 1 mL of the inoculum were flooded with a warm agar medium (46–48 °C) in a 15–20 mL volume and gently mixed. After the medium’s solidification, the dishes were incubated. The parameters of the experiments are presented in Table 3. After incubation, CFU values were determined visually.

The determination of viable bacteria was calculated according to Equation (1):

(1) $$N=\left(100\times C\times D\times V\right)/A$$

wherein N is the number of CFU/sample; C is the average number of CFU; D is the dilution factor; V is the volume of the neutralizer, mL; and A is the area of the release liner, mm^2^. All experiments were designed and carried out according to JIS Z 2801:2000 ISO 22196:2007, ASTM E 1054 [37].

#### 2.4.6. Test of Validity of the Results

The logarithmic value of viable cells in the culture control must meet the condition in Equation (2):

(*L_max_* − *L_min_*)/*L_mean_* ≤ 0.2 (2)

wherein *L_max_* is the decimal logarithm of the maximum number of CFU; *L_min_* is the decimal logarithm of the minimum number of CFU; and *L_mean_* is the base 10 logarithm of the average CFU.

### 2.5. Antiviral Activity Tests of Polymer Composite with Triclosan

#### 2.5.1. Sterilization of Samples

A sample of the triclosan-containing polymer composite was placed in the working chamber of a biological safety cabinet (BSC) and quartz-treated for 15 min, after which it was treated with 96% ethanol and burned. Control Petri dishes, 3 pieces each, were wrapped in kraft paper, autoclaved, packed in quartz in the working chamber of the SBB, and opened immediately before applying the virus-containing solution to them.

#### 2.5.2. Application of Viruses and Flushing of Virus-Containing Material

The human influenza virus A/ZKO/46/19 at the initial concentration, 100 μL in volume, was applied to the working surface of composite tiles and to the inner surface of Petri dishes. The duration of virus exposure on the surface of the composite tiles was 10, 30, and 60 min. Sterile Petri dishes were used as negative controls, on which the influenza A/WKO/46/19 virus was also applied for 10, 30, and 60 min. To control the growth of the virus, 6 × 10-fold dilutions of the initial virus-containing solution were used. The virus and swabs from the samples were cultured in the allantoic cavities of 9–10-day-old chicken embryos. The presence of the virus in the allantoic fluid was determined using RGA. All procedures were performed under sterile conditions. Avian coronavirus infectious bronchitis virus (IBV) was applied on the test samples and control Petri dishes according to the same scheme and method as in the case of influenza virus A/WKO/46/19 (Table 4).

After the expiration of the incubation period, 900 μL of sterile PBS was evenly applied to the working surface of the composite triclosan-containing tiles and control Petri dishes and incubated for 1 min, after which they were collected in sterile vials. All procedures were performed under sterile conditions.

#### 2.5.3. Sample Preparation before Culturing in Chicken Embryos

Influenza A/ZKO/46/19 swabs were diluted in sterile PBS until 6 × 10-fold dilutions were obtained, considering the swab itself to be one 10-fold dilution. To control growth, the original sample of influenza virus A/WKO/46/19 was also diluted to obtain six 10-fold dilutions. All six dilutions were injected into the allantoic cavities of developing chicken embryos. IBV washes were diluted in sterile PBS until 3 × 10-fold dilutions were obtained, considering the wash itself to be one 10-fold dilution. To control growth, the original sample of IBV was diluted to obtain three 10-fold dilutions; all three dilutions were injected into the allantoic cavities. Each experiment was performed in 4 replicates (4 embryos per sample).

#### 2.5.4. Virus Culture in Developing Chicken Embryos

For the cultivation of influenza virus or IBV in chicken embryos, the test materials, after their primary processing, were injected (0.2 mL) into the allantoic cavities of 9–11-day-old chicken embryos. Infection was carried out in a sterile box through the side surface of the shell, directing the needle into the allantoic cavity, after making a puncture in the shell above the air chamber. Infected embryos were incubated in a thermostat for 48 h at 36–37 °C (for influenza viruses) and 96 h at 33–34 °C (for IBV). After incubation, the embryos were stored overnight at +4 to +8 °C. The allantoic fluid was collected in separate test tubes.

The presence of the influenza virus in the allantoic fluid was determined in a hemagglutination test (HA). In contrast, the presence of IBV was determined by the yield of the amplification product during real-time polymerase chain reaction (RT-PCR). The infectious titer of IBV was calculated according to the method of Reed and Mench.

#### 2.5.5. Preparation of 0.5% Suspension of Chicken Erythrocytes

The blood of roosters was collected from the heart or axillary vein. Freshly obtained blood was placed into a vial with one of the anticoagulants (Alsever’s solution, 5% sodium citrate solution). Blood defibrination was carried out immediately by intensive shaking of the vial for 5–7 min at a temperature of (20 ± 2) °C until fibrin fibers fell out. Defibrinated blood was filtered through 4 layers of gauze and then washed out with PBS three times by centrifugation at 1500 rpm for 5 min, and then the supernatant was removed. A 0.5% suspension of chicken erythrocytes by volume was prepared from the sediment, taken as 100%, by adding sterile PBS.

#### 2.5.6. Determination of Hemagglutinating Antigen Titer (RGA)

PBS was added to each well of one row of a microplate in a volume of 50 µL. Then, 50 μL of antigen at a dilution of 1:10 was added to the first well, and then titration was carried out according to the principle of two-fold dilution. Next, 50 µL was removed from the last well. Then, 50 µL of a 0.5% erythrocyte suspension was added to each well. Finally, the contents of the wells were mixed by shaking and left at room temperature for 40–45 min until erythrocytes settled in the control.

The antigen titer, or one agglutinating unit (AU), was taken as the highest dilution of the antigen, which causes the pronounced agglutination of erythrocytes. The determination of the antigen titer was accompanied by the setting of a negative control for the absence of spontaneous agglutination of erythrocytes. Several panel wells served as negative controls, into which sterile PBS was added instead of the antigen.

#### 2.5.7. Isolation of Total RNA from Allantois

Total RNA was isolated using the Pure Link Viral DNA/RNA kit (Invitrogen, Waltham, MA. USA) according to the manufacturer’s protocol. Reverse transcription to obtain cDNA was conducted by M-MLV reverse transcriptase (Invitrogen, Waltham, MA, USA) in 10 μL of a reaction mixture containing 4 μL of RNA, 2 μL of water (DEPC treated), 2 μL of 5× reverse transcriptase buffer (Invitrogen, Waltham, MA, USA), 0.5 µL of gene-specific primer, 0.5 µL of 10 mM dNTP mixture, and 1 µL of M-MLV (20 units/µL). The reaction was carried out at 37 °C for 50 min, followed by reverse transcriptase deactivation at 70 °C for 15 min.

#### 2.5.8. RT-PCR Setup

RT-PCR was performed in 20 µL of the reaction mixture (SybrGreen Master Mix—10 µL; forward gene-specific primer 5 µM—0.5 µL; reverse gene-specific primer 5 µM—0.5 µL; ROX solution (to normalize the fluorescence intensity of intercalating dsGreen type dyes)—0.04 µL; deionized water—5.46 µL). The PCR mixture was added to the plate wells at 16.5 μL, taking care not to allow air bubble formation. cDNA aliquots (3.5 µL each) were added to predetermined plate wells and sealed with optical film. The plate (strip) was centrifuged to collect all drops from the walls. A plate (strip) was installed in the cycler, marking the location and characteristics of the samples in the program and choosing a working dye. PCR was carried out at 95 °C—5 min, 94 °C—1 min, 50 °C—1 min, 72 °C—1 min, 40 cycles. The presence of specific nucleic acids was detected by using real-time spectrophotometry during the amplification reaction.

#### 2.5.9. Determination of EID50 of IBV According to the Method of Reed and Mench

The titration of the virus in test-tube cultures and animals involves determining the dose at which the effect of the virus is manifested in 50% of the test objects (chicken embryos), the so-called EID50. The calculation of EID50 is carried out according to the f total cumulative method of Reed and Mench. This method is based on the logical premise that a chick embryo showing signs of infection (in the case of IBV, the presence of an amplification product in allantois samples during RT-PCR) after the injection of any dilutions of the virus will show signs of infection at any lower dilutions.

Take the dilution at which 50% of the infection result was recorded and then calculate the value of *X*, which must be added to the dilution immediately below 50% of the dose (in lg). *X* is calculated using Equation (2):

(3) $$X=\frac{A-50}{A-B}$$

where A is the percentage of infected embryos at a dilution just below the target 50% dose, and B is the percentage of infected embryos at a dilution just above the target 50% dose.

## 3. Results and Discussion

### 3.1. Preparation of Polymer Composites with Triclosan

**Figure 3:**
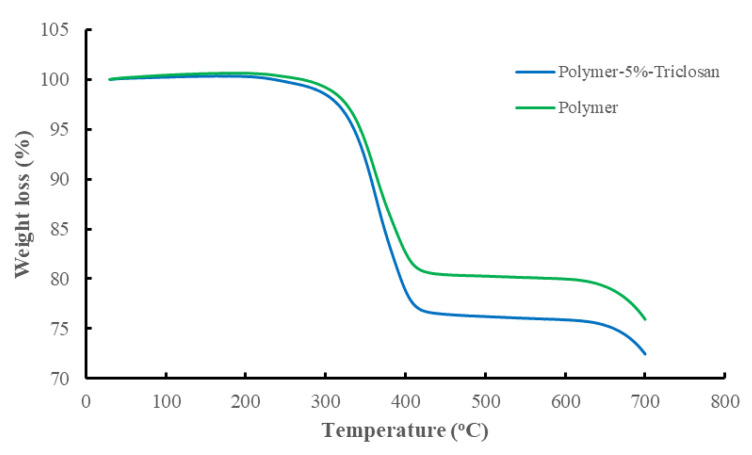
Thermo-analysis of the reference and 5 wt.% triclosan-loaded polymer composites.

As described in our previous study [36], wherein the same reference and 5 wt.% triclosan-loaded polymer composites were used, the difference in the chemical compositions of the reference and 5 wt.% triclosan-loaded polymer composites revealed a significant alteration in the elemental composition with the antibacterial agent triclosan, wherein 1.67 wt.% chlorine is identified. The latter molecule contains three atoms of chlorine connected to aromatic rings. These account for one-third of the total weight of the triclosan molecule, which closely matches the chemical analysis of the 5 wt.% triclosan-loaded polymer composite (Table 5). Further evidence of the successful modification with the antibacterial agent is the thermogravimetric analysis. The results of thermo-analysis revealed around a 5% weight loss difference between the reference and triclosan-loaded polymer composites, which is due to the molecule of aromatic nature also taking part in carbonization (Figure 3).

**Figure 4:**
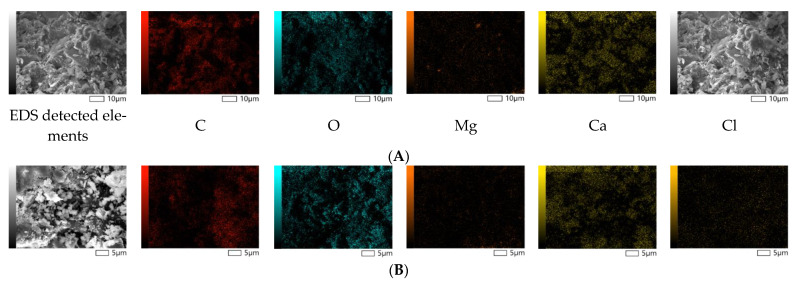

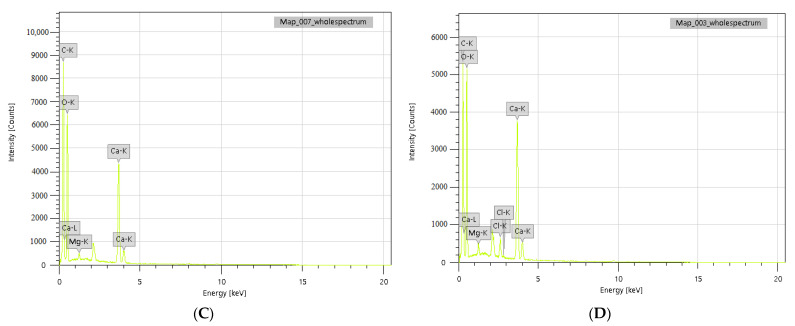
SEM-EDS micrographs of the reference (**A**,**C**) and 5 wt.% triclosan-loaded (**B**,**D**) polymer composites.

The morphological analysis of the reference and polymer composites demonstrates the non-porous structure of the samples, as shown in Figure 4. These characteristics are favorable for producing sanitary products to reduce the formation of the humid environment required for the growth of viruses and bacteria.

The reference and polymer composite samples were also studied using the EDS method, indicating the contents of the main elements in the selected spots. The major fractions of both the reference and modified polymer composites consist of calcium, oxygen, carbon (97.31 wt.% and 99.49 wt.%), and a trace amount of magnesium. The most important finding is the detection of chlorine in the amount of 1.92 wt.%, which agrees with the chemical composition analysis, as presented in Table 6. Taking into account the molecular weight of chlorine within the organic structure of triclosan, we may tentatively calculate the theoretical mass of chlorine from the initially loaded 5 wt.% triclosan, which corresponds to 1.83 wt.%. This value is comparable to the average elemental analysis results (1.79 wt.%); therefore, we may assume that almost all loaded triclosan is homogeneously distributed within the polymer matrix.

The porosimetric analysis of the reference and polymer composites with 5 wt.% triclosan showed a non-porous microstructure with specific surface areas of less than 1.92 m^2^/g. A decrease in the specific surface area of the 5 wt.% triclosan-loaded polymer composite can be attributed to the addition of an antibacterial agent that might also serve as a binder to make the polymeric structure denser.

### 3.2. Antimicrobial Activity of Polymer Composites with Triclosan

The purpose was to evaluate the preservation of the antimicrobial efficacy of composite samples under the influence of certain physicochemical factors. The experimental samples were exposed to sunlight and UV irradiation in order to simulate natural conditions. In addition, the effects of acidic and alkaline environments on the antimicrobial activity of the samples were also studied.

The prototypes, depending on the impact factor, were divided into three experimental groups: (1) the effect of sunlight; (2) the effect of UV radiation; (3) the influence of the pH of the medium. Composites were subjected daily to appropriate processing. Tests of the maintenance of antimicrobial efficacy were carried out after 7 and 14 days and then monthly after daily treatment. The results of validity tests for each studied experimental condition are shown in Table 7.

The antimicrobial activity of composite samples was studied with the addition of triclosan (5%) against *S. aureus* 6538-P, and the contact time was 2 h. Composite samples of identical composition without the addition of triclosan were used as negative controls. Control samples were not exposed. Experiments with control and experimental samples were conducted in parallel under the same conditions. The results of antimicrobial activity testing under different physicochemical conditions are presented in Table 8. The conducted studies indicate a high bactericidal effect of the triclosan-containing composite sample. The obtained values indicate 100% antimicrobial activity preservation against *S. aureus* 6538-P in all experimental groups after a 2 h exposure.

Generally, polymeric materials are classified into four categories based on the origin of the antimicrobial properties: polymers with inherent self-antibacterial activity (a), modified polymers with antibacterial properties (b), and polymeric composites incorporated with organic (c) and inorganic (d) antimicrobial compounds [38]. There are several related studies on polymer composites incorporated with triclosan as an antimicrobial agent. The latter molecule was studied using conformational computational chemistry in order to understand the behavior of the alternating ether bond rotations within the polymer matrix [39]. Polystyrene incorporated with triclosan using a melt-mixing approach was studied to provide persistent antibacterial action on the surface against resistant strains of *E coli* and *B. thuringiensis*, which showed significantly improved inhibitory activity within 20 h of exposure [40]. More recently, polymeric nanogels were developed to deliver triclosan with a hydrophobic moiety to a target site in order to substantially enhance the antimicrobial efficiency against planktonic bacteria [41]. The results of this study are essential, as triclosan-loaded polymeric composites have not been studied in similar conditions as an antibacterial material with a non-porous microstructure for the coating or manufacturing of construction or sanitary furniture.

Furthermore, the current study included extensive research on aging effects under various experimental conditions and examined the triclosan-loaded polymeric composites as a potential antiviral surface-coating material. Therefore, this study could be a valuable reference for future research on polymeric materials with both antibacterial and antiviral activities. Previously studied materials include the natural polymer chitosan with embedded triclosan as an antimicrobial agent for adhesive resin against *S. mutans* [42], a blend of polyethylene and triclosan against *E. coli* and *Kl. pneumoniae* and against *S. aureus* [30], and composites of triclosan-loaded poly(ε-caprolactone)/polylactic acid nanoparticles against *S. aureus* and *E.coli* [31].

### 3.3. Antiviral Activity of Polymer Composite with Triclosan

#### 3.3.1. Study of the Influence of the Surface of a Composite Triclosan-Containing Material on the Infectious Activity of the Influenza Virus

Triclosan was selected for its efficiency not only as an antimicrobial agent but also as a compound that shows some antiviral activity. Thus, the reduction in virus infectivity after a 30 s contact time with triclosan at 0.05% illustrated 4.8 log_10_TCID_50_/mL, comparable to the action of 0.21% Sodium hypochlorite [43]. Another study has shown that 0.6% triclosan in soap has log_10_TCID_50_ values of 0.25 and 0.5 for 0.5 and 2 min contact times, comparable to 3% hydrogen peroxide [44]. The determination of virucidal activity is one of the main approaches to assessing the effectiveness of antiviral drugs and materials. Therefore, the virucidal activity of a self-disinfecting composite triclosan-containing material was studied using a human influenza virus model, epidemic strain of 2019—A/ZKO/46/1.

When determining the presence of the virus in the selected allantois using hemagglutination, zero RGA titers were recorded in 5 and 6 10-fold dilutions of the sample with a 30 min exposure on composite tiles and 3–6 dilutions of the sample with a 60 min exposure on composite tiles. In all dilutions of samples washed from Petri dishes, there were no zero titers of RHA, indicating the presence and reproduction of the virus in these samples (Table 9).

**Figure 5:**
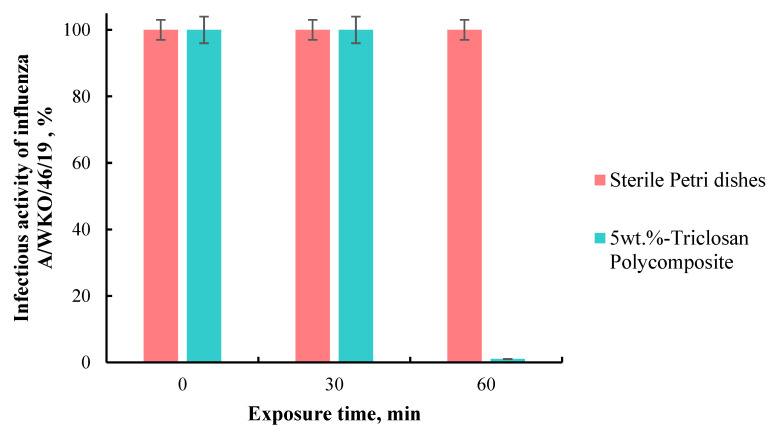
The infectious activity of influenza A/WKO/46/19 virus after 60 min of incubation on sterile Petri dishes and polymer composite.

As a result, it was shown that the incubation of the influenza A/WKO/46/19 virus on self-disinfecting triclosan-containing tiles for 30 and 60 min reduces its infectious activity by 2 and 4 lg, respectively, or to 1 and 0.01% of the original (Figure 5).

Thus, it was found that the contact of the human influenza virus with tiles consisting of a composite triclosan-containing material for 60 min decreases its infectious activity by 99.99%, comparable to the effect of quartz. Therefore, this material can be effectively used for disinfection against the influenza virus.

#### 3.3.2. Study of the Influence of the Surface of a Composite Triclosan-Containing Material on the Infectious Activity of IBV

The virucidal activity of the composite triclosan-containing material was studied using the IBV model, strain H-120, Massachusetts serotype, belonging to the Coronaviridae family. The exposure of the virus to the surface of self-disinfecting tiles lasted for 10, 30, and 60 min. Sterile glass Petri dishes were used as negative controls, on which the IBV virus was also applied for 10, 30, and 60 min. To test the growth of the virus, 1-, 2-, 3-, and 10-fold dilutions of the original vaccinated solution were used. The virus and swabs from the samples were cultured in the allantoic cavities of 10-day-old chicken embryos. The presence of the virus in the allantoic fluid was determined by the yield of the amplification product during RT-PCR. The level of infectious activity of the virus was calculated by the method of Reed and Mench.

**Figure 6:**
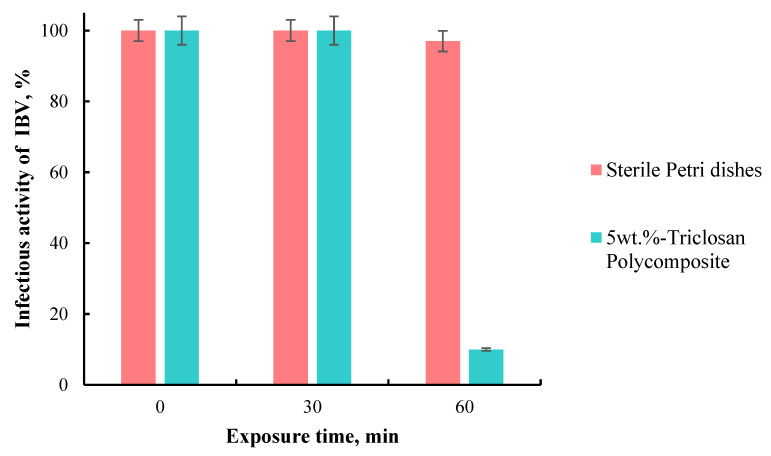
Infectious activity of IBV after 60 min of incubation on sterile Petri dishes and polymer composite.

As a result, it was shown that the incubation of the IBV virus on the surface of self-disinfecting polymer tiles for 30 and 60 min reduces the infectivity titer by 0.5 and 1 lg, respectively, i.e., up to 30 and 10% of the original. However, 10 min of virus contact with polymer tiles did not reduce its infectivity. Furthermore, exposure to the virus on sterile Petri dishes for 10, 30, and 60 min barely decreases its infectivity (Figure 6).

Thus, it was found that the contact of IBV with the surface of a composite triclosan-containing material within 60 min reduces its infectious activity by 90%. Therefore, this material can be used for combating and preventing coronavirus infections. These data are in agreement with the previous confirmation of the antiviral activity of triclosan; the test results illustrate that the rate of decline in the virus titer is 3 log for triclosan-immobilized cotton textiles, while the untreated cotton textile causes no decline [24,45].

## 4. Conclusions

Triclosan-loaded polymer composites produced through an automated system, according to the findings, possess a non-porous structure that is further improved by loading an antibacterial agent into the microstructure, making it more solid. It has been proven that a polymer composite with the addition of triclosan (5%) under in vitro conditions has a high antimicrobial effect against the *S. aureus* 6538-P strain (death of 100% of cells). Furthermore, an in vitro study under the influence of physicochemical factors such as sunlight, UV radiation, and medium pH changes revealed the preservation of the bactericidal activity of the test samples against *S. aureus* 6538-P after 7 and 14 days, as well as after 1 and 2 months of daily exposure. Thus, it was determined that the antimicrobial activity of triclosan-containing samples is maintained after 2 months of daily exposure to external factors that mimic natural conditions.

In addition, it was revealed that the contact of the human influenza virus, strain A/WKO/46/19, with the surface of a self-disinfecting composite triclosan-containing material for 60 min at room temperature reduces its infectious activity by 99.99%, and contact of the infectious IBV (strain “H-120” serotype Massachusetts) with the surface of the composite triclosan-containing material for 60 min at room temperature reduces its infectious activity by 90%. Thus, it was shown that a self-disinfecting composite triclosan-containing material could be an effective tool for combating and preventing epidemics of the influenza virus and coronavirus.

## Figures and Tables

**Table 1:** Polymers loaded with various antimicrobial agents.

Polymeric Matrix	Antimicrobial Agent	Target Microorganism	Ref.
Polyethylene glycol	Sulfamethoxazole and trimethoprim	*S. aureus*; *E. coli*	[26]
Zwitterionic PTMAEMA-co-PSPE polymer	Poly(styrenesulfonate), quaternary ammonium, H_2_O_2_ enzyme	*S. aureus*	[27]
Phenylboronic acid polymer brushes	Vancomycin	*S. aureus, S. epidermidis*	[28]
Poly(glycidyl methacrylate) brushes	Quaternized polyethylenimine	*S. aureus*	[29]
Polyethylene	Triclosan	*E. coli; Klebsiella pneumoniae; S. aureus*	[30]
Poly(ε-caprolactone)/polylactic acid nanoparticles	Triclosan	*S. aureus; E. coli*	[31]
Polyamide 11/polymeric biocide polyhexamethylene guanidine	Dodecylbenzenesulfonate	*E. coli*; *Bacillus subtilis*	[32]
Polyurethane	Ciprofloxacin	*Pseudomonas aeruginosa*	[33]
Polymer of oligoethylene glycol, cationic primary amine, and hydrophobic ethylhexyl	Nitric oxide	*P. aeruginosa*	[34]

**Table 2:** The manufacturing of the reference and triclosan-loaded polymer composites.

Sample Name	Composition of Samples	Weight %
Reference polymer composite	MEKP	2
	Unsaturated polyester resin	28
	CaCO_3_	70
5 wt.% triclosan-loaded polymer composite	MEKP	2
	Triclosan	5
	Unsaturated polyester resin	28
	CaCO_3_	65

**Table 3:** The incubation parameters used for antimicrobial studies.

Surface Type of Sample	Chemical Composition
Sample	Unsaturated ester of orthophthalic acid, calcium carbonate, and methyl ethyl ketone peroxide with triclosan content of 5 wt.%; 8 cm in diameter
Reference sample	Unsaturated ester of orthophthalic acid, calcium carbonate, and methyl ethyl ketone peroxide without triclosan; 8 cm in diameter
Release liner size	40 mm × 40 mm
Culture medium	Nutrient agar, pH 7.4 ± 0.2 Incubation time and conditions: 37 ± 1 °C; 18–24 h
Inoculum preparation medium	1/50 Nutrient broth, pH 7.4 ± 0.2 Inoculum concentration—2.5–10.0 × 10^5^ CFU/mL Amount of applied inoculum—0.4 mL
Contact time and incubation conditions	5, 15, and 30 min and 1, 2, and 4 h 37 ± 1 °C; humidity: ≥90%
Neutralizer	Tryptic soy broth with lecithin and tween-80, pH 6.8–7.2
Medium for counting CFU	Nutrient agar, pH 7.4 ± 0.2 Incubation time and condition—37 ± 1 °C; 40–48 h

**Table 4:** Scheme of applying a virus-containing material to the studied samples of self-disinfecting tiles.

Sample Number	Incubation Time, min	Strain of Virus
No. 1	10	Influenza virus (strain A/WKO/46/19)
No. 2	30	
No. 3	60	
No. 4	10	Coronavirus, IBV (strain “H-120” serotype Massachusetts)
No. 5	30	
No. 6	60	

**Table 5:** The chemical compositions of polymer composites (wt.%).

Chemical Elements	Polymer Composite	Polymer Composite with 5 wt.% Triclosan
Mg	0.40	0.39
Ca	48.99	47.61
S	0.02	0.02
Fe	0.01	0.01
Cl	-	1.67

**Table 6:** The elemental analysis of the polymeric composites on SEM-EDS (in wt.%).

Sample	Ca	Mg	C	O	Cl
Polymer composite	21.20	0.50	34.09	44.20	-
Polymer composite with 5 wt.% triclosan	22.89	0.77	29.97	44.45	1.92

**Table 7:** The results of testing the validity of the experiments to assess antimicrobial activity.

Exp. No.	Strain	Average Number of CFU	Average Value, Log10	Value of L_max_/L_min_	Value of (L_max_ − L_min_)/L_mean_
1 (after 7 days)	*S. aureus* 6538-P	4.25 × 10^3^	3.628	3.615/3.641	0.007
2 (after 14 days)		3.34 × 10^3^	3.524	3.495/3.552	0.016
3 (after 1 month)		3.38 × 10^3^	3.528	3.503/3.552	0.014
4 (after 2 months)		4.00 × 10^3^	3.602	3.574/3.628	0.015

**Table 8:** The results of the antimicrobial activity of the polymer composite with triclosan subjected to aging effects.

Exp. No.	Control	Sunlight	Antimicrobial Activity, Log10/% *	UV	Antimicrobial Activity, Log10/% *	Acidic Phase	Antimicrobial Activity, Log10/% *	Basic Phase	Antimicrobial Activity, Log10/% *
		Avg. Number of CFU/Avg. Log10 Value		Avg. Number of CFU/Avg. Log10 Value		Avg. Number of CFU/Avg. Log10 Value		Avg. Number of CFU/Avg. Log10 Value	
1 (after 7 days)	3.59 × 10^3^/3.556	0.06/−1.204	4.760/100%	0.06/−1.204	4.760/100%	0.06/−1.204	4.760/100%	0.06/−1.204	4.760/100%
2 (after 14 days)	2.66 × 10^3^/3.424	0.06/−1.204	4.628/100%	0.06/−1.204	4.628/100%	0.06/−1.204	4.628/100%	0.06/−1.204	4.628/100%
3 (after 1 month)	4.09 × 10^3^/3.612	0.06/−1.204	4.816/100%	0.06/−1.204	4.816/100%	0.06/−1.204	4.816/100%	0.06/−1.204	4.816/100%
4 (after 2 months)	3.47 × 10^3^/3.540	0.06/−1.204	4.744/100%	0.06/−1.204	4.744/100%	0.06/−1.204	4.744/100%	0.06/−1.204	4.744/100%

* Conversion from CFU values of the control sample.

**Table 9:** Determination of the presence of influenza virus A/WKO/46/19 in allantois using RGA.

Sample	Titer of Viral Particles in 1 mL before Infection	Incubation Time, mins	Average Titer RGA	Standard Deviation
Petri dishes	10,000	10	341.3	147.8
		30	256.0	0
		60	213.3	73.9
	1000	10	256.0	0
		30	213.3	73.9
		60	213.3	73.9
	100	10	256.0	0
		30	213.3	73.9
		60	213.3	73.9
	10	10	256.0	0
		30	213.3	73.9
		60	170.7	73.9
Polymer composites	10,000	10	213.3	73.9
		30	170.7	73.9
		60	0	0
	1000	10	170.7	73.9
		30	32.0	32
		60	0	0
	100	10	128.0	0
		30	0	0
		60	0	0
	10	10	64.0	0
		30	0	0
		60	0	0
Influenza virus A/WKO/46/19	10,000	×	341.3	147.8
	1000	×	341.3	147.8
	100	×	213.3	73.9
	10	×	213.3	73.9

## Data Availability

The data presented in this study are available on request from the corresponding author.
